# Landmark-Based Inertial Navigation System for Autonomous Navigation of Missile Platform

**DOI:** 10.3390/s20113083

**Published:** 2020-05-29

**Authors:** Donghui Lyu, Jiongqi Wang, Zhangming He, Yuyun Chen, Bowen Hou

**Affiliations:** 1 College of Liberal Arts and Science, National University of Defense Technology, Changsha 410073, China; ldh_gfkd@163.com (D.L.); hezhangming@nudt.edu.cn (Z.H.); houbowen12@nudt.edu.cn (B.H.); 2 School of Mathematics and Big Data, Foshan University, Foshan 528225, China; kasineya@sina.com; 3 Beijing Institute of Spacecraft System Engineering, China Academy of Space Technology, Beijing 100094, China

**Keywords:** missile platform, landmark observation, inertial system, observability analysis, navigation accuracy

## Abstract

As a new information provider of autonomous navigation, the on-orbit landmark observation offers a new means to improve the accuracy of autonomous positioning and attitude determination. A novel autonomous navigation method based on the landmark observation and the inertial system is designed to achieve the high-accuracy estimation of the missile platform state. In the proposed method, the navigation scheme is constructed first. The implicit observation equation about the deviation of the inertial system output is derived and the Kalman filter is applied to estimate the missile platform state. Moreover, the physical observability of the landmark and the mathematical observability of the navigation system are analyzed. Finally, advantages of the proposed autonomous navigation method are demonstrated through simulations compared with the traditional celestial-inertial navigation system and the deeply integrated celestial-inertial navigation system.

## 1. Introduction

The navigation system plays an important role in the actual application of the missile. Inertial Navigation System (INS), Global Navigation Satellite System (GNSS), and Celestial Navigation System (CNS) [1] are the three most commonly used missile navigation systems at present.

INS uses an accelerometer and a gyroscope to measure the line motion and angle motion of the carrier platform. Through the navigation solution, parameters such as speed, position, and attitude of the carrier platform relative to a reference coordinate system can be determined [2]. INS has the advantages of better concealment, stronger autonomy, and higher short-term accuracy. However, the output error of INS accumulates with time. Therefore, INS cannot be used in the missile platform alone.

GNSS can provide rich state information of the carrier platform such as the position and the speed [3]. However, GNSS is semi-autonomous. Influenced by the complex space conditions such as uncertain electromagnetic interference and possible signal occlusion as well as the inherent vulnerability of navigation signals, GNSS’s availability and service ability are uncertain to a large extent [4]. It may also be difficult for the receiver to track GNSS signals because of the large Doppler frequency shift of GNSS signals resulting from high dynamic characteristics of the missile platform.

CNS determines the state of the carrier platform by observing celestial bodies and has the advantages of stronger autonomy and concealment. However, the output of CNS is low-frequency and is easily affected by the external environment.

Because of shortcomings of each single navigation system, the integrated navigation system has become a key means to improve accuracy and reliability of autonomous navigation by complementing advantages of different navigation schemes. Among different integrated navigation systems, the Celestial-Inertial Navigation System (CINS) composed of CNS and INS has become the most commonly used one in the missile platform [5,6] due to its strong autonomy and the concealment of the CNS and INS.

CINS can correct the accumulation error of INS through starlight information [5]. There are two modes in the combination of INS and CNS: the loose one and the tight one. In the loose combination mode, CINS obtains the state estimation by fusing the independent output of INS and CNS [7,8,9]. However, two or more observable stars are needed at the same time for the loosely combined CINS to carry out the filter process. Meanwhile, the complementary characteristics of INS and CNS are not fully utilized in the loose combination mode. Therefore, the tightly combined CINS is constructed, In which CINS directly fuses the INS output with the astronomical observation to estimate the missile platform state even there is only one observable star. In addition, the tightly combined CINS can improve the navigation accuracy by avoiding the twice use of the random model [10,11,12,13].

However, due to star sensor’s inability to suppress the output error of the accelerometer, both the loosely combined and the tightly combined CINS are faced with the divergence of the position estimation error. To improve the position estimation accuracy of CINS, different efforts have been made. For example, CINS based on the star refraction [6,14] could obtain the horizontal reference by sensing more than three star refraction angles. Further, the position of the missile platform could be estimated. However, the accurate model of atmospheric density that the method relied on was difficult to be established. At the same time, it is difficult to meet the condition of more than three refracting stars. Wang et al. [13] achieved the high-accuracy estimation of all aircraft states by adding the complete weightlessness constraint in the dynamic model of the automated transfer vehicle. However, the constraint was only applicable to the high-orbit vehicles and could not completely suppress the divergence of the position estimation error. Yang [15] et al. estimated the accelerometer bias by sensing the missile altitude with a barometer. However, due to the inevitable error of the atmospheric density model, the accuracy of this method was also limited. Ning et al. [12] focused on the INS rotary modulation technology, through which the gyro drift and the acceleration deviation could be compensated. However, the technology inevitably increased the energy consumption of the navigation system and the inaccurate rotation control may also reduce the accuracy of the state estimation.

In recent years, the visual sensor has widely been used in navigation systems due to the rapid development of the image processing technology and advantages of the visual sensor in weight, cost, and power consumption. The visual sensor can be applied to the space-target navigation in three ways. The first method determines the carrier platform state by comparing the measured image with the stored one [16]. The second obtain state estimation by sensing the direction from the carrier platform to the landmark [17,18]. The third one calculates the motion of the carrier platform according the continuous images taken by the camera [19].

Among the three means, the method based on the landmark, i.e., the second method, has been widely used in many fields such as autonomous landing and satellite orbit determination because of its advantages of bounded navigation parameter error and more simple calculation [20]. For instance, NASA obtained the attitude estimation of the lander by matching landmarks taken by the camera with the reference landmarks in the ALHAT program [21,22]. Xu et al. extracted the known landmarks and the unknown landmarks from images taken by the lander to establish the measurement equation, through which the INS output deviation could be corrected and the high-accuracy estimation of the position and the attitude could be given [23]. Hou et al. established an autonomous navigation scheme based on the observation of Mars landmarks and got higher orbit determination accuracy of Mars orbiting [24]. Youngsun and Dong-Hwan built an integrated navigation system through landmarks and inertial devices. Even when the number of the visible landmarks is small, the system could still get much more reliable output of position, speed, and attitude [25].

Therefore, in view of the CINS’s difficulties in the position estimation and advantages of the landmark-based navigation method, the landmark-based method is combined with INS to form an autonomous navigation system for the missile platform. Through the proposed system, named Landmark-based Inertial Navigation System (LINS), the paper attempts to achieve higher accuracy estimation both of the attitude and the position for the missile platform without adding additional sensors. The main contributions of this paper include the following.

1. The construction of the autonomous navigation scheme: The implicit observation equation of the INS output deviation is established based on the landmark observation and the corresponding coordinates estimation (calculated according to INS output and the known landmark location). Combining the constructed observation equation and the ballistic error propagation model, the position and the attitude of the missile platform are estimated through Kalman filter.
2. The analysis of the observability: From two aspects—the physical observability of landmarks and the mathematical observability of the navigation system—the observability of the proposed system is analyzed. The paper proves that the proposed navigation system is completely observable mathematically when the number of observable landmarks is greater than 1.
3. The realization of the comparative simulation experiments: Compared with the traditional CINS and the deeply integrated CINS, the simulation experiments prove that LINS can greatly improve the accuracy of position estimation while maintaining the attitude estimation accuracy.

It should be pointed out that although the mind of using visual sensors to aid the navigation of INS adopted by this paper is the same as that of other literatures, see, e.g., [23,25], the state transition equation and the observation equation used in this paper are different from those in other articles due to the change of the navigation environment. In the construction of the observation equation, the derivation of equations about error propagations of position and attitude are done by authors. The observability analysis and simulation design given in this paper can also be recognized as innovative.

In view of above research content, the paper will be carried out in the following order. In Section 2, the navigation scheme of LINS is designed. The propagation equation of ballistic error, the implicit observation equation, as well as the state estimation process of the missile platform are given. Section 3 analyzes the physical observability of landmarks and the mathematical observability of the proposed navigation system. In Section 4, advantages of LINS are proved by the comparative simulation experiments. Finally, Section 5 draws our conclusion.

## 2. LINS Navigation Scheme and State Estimation Process

In this section, the autonomous navigation scheme of LINS is designed first. Next, the propagation model of the ballistic error, i.e., the state transition equation of the proposed navigation system, is introduced. Then, the implicit observation equation about the INS output deviation is derived. Finally, based on the existing state transition equation and the observation equation, the Kalman filter is applied to the state estimation. Meanwhile, the estimation process of missile platform state is also given.

For the sake of simplicity, the coordinate systems used in the missile navigation are given as follows, the launch-point inertial coordinate frame (li-frame), the missile body coordinate frame (b-frame), and the sensor coordinate frame (s-frame). The specific definition of the coordinate system can be seen in [15]. At the same time, we assume that b-frame and s-frame have the same origin.

### 2.1. Autonomous Navigation Scheme of Missile Platform

As mentioned above, the star sensor used in CINS cannot provide the position information of the carrier platform. Moreover, because of the infinite distance between the missile platform and the star, the position change of the missile has little influence on the observation coordinate of the star. That is to say, the star observation contains little position information of the missile platform.

Different from stars, the distance between the landmark and the missile is limited. Both changes of the position and of the attitude can cause great impact on the observation coordinates of the landmark. In other words, the landmark observation contains more abundant state information of the missile platform than the star observation. Therefore, the landmark observation may provide a new means to improve the accuracy of autonomous orbit determination as a novel information source of autonomous navigation.

**Figure 1:**
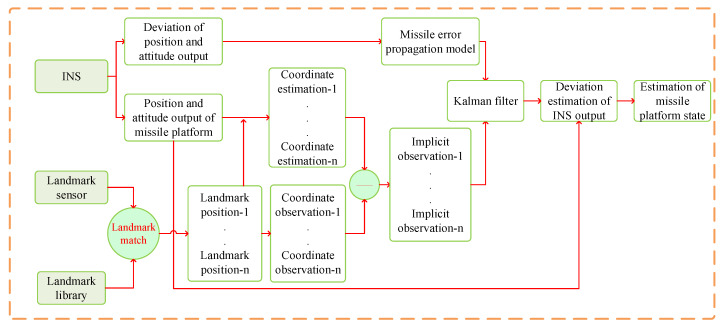
Autonomous navigation scheme of Landmark-based Inertial Navigation System (LINS).

Based on the above basic idea, the navigation scheme of LINS is constructed in Figure 1 to get the high-accuracy estimation both of the position and the attitude for the missile platform.

As shown in Figure 1, the autonomous navigation scheme obtains coordinates of landmarks with known positions through the landmark matching, and also gets the coordinate estimation of landmarks with INS output. Based on the difference between the landmark observation and the landmark estimation, the implicit observation equation of the INS output deviation can be established. Combined with the ballistic error propagation model, the Kalman filter is applied to the estimation of the INS output error. Furthermore, by the feeding the deviation estimation back to the INS output, the missile platform state is finally estimated.

### 2.2. Ballistic Error Propagation Equation

In this paper, we select li-frame as the navigation frame. All of the following variables are expressed in this frame. Take $X\left(t\right)={\left[\phi {\left(t\right)}^{T},\delta V{\left(t\right)}^{T},\delta r{\left(t\right)}^{T},\varepsilon {\left(t\right)}^{T},\nabla {\left(t\right)}^{T}\right]}^{T}$ as the system state vector, where $\phi \left(t\right)={\left[\phi_{x}\left(t\right),\phi_{y}\left(t\right),\phi_{z}\left(t\right)\right]}^{T}$ is the misalignment angle of the missile platform, i.e., the attitude estimation error of the missile platform; $\delta V\left(t\right)={\left[\delta V_{x}\left(t\right),\delta V_{y}\left(t\right),\delta V_{z}\left(t\right)\right]}^{T}$ and $\delta r\left(t\right)={\left[\delta x(t),\delta y(t),\delta z(t)\right]}^{T}$ are the estimation errors of the speed and the position for the missile platform, respectively; $\varepsilon \left(t\right)={\left[\varepsilon_{x}\left(t\right),\varepsilon_{y}\left(t\right),\varepsilon_{z}\left(t\right)\right]}^{T}$ represents the angle drift resulting from gyro bias; and $\nabla \left(t\right)={\left[\nabla_{x}\left(t\right),\nabla_{y}\left(t\right),\nabla_{z}\left(t\right)\right]}^{T}$ is accelerometer’s constant offset.

The ballistic error propagation equation is as follows,

(1) $$\dot{X}\left(t\right)=F\left(t\right)X\left(t\right)+G\left(t\right)W\left(t\right),$$

where F(t) is the process input matrix and

(2) $$F\left(t\right)=\left[\begin{array}{ccccc} 0_{3\times 3} & 0_{3\times 3} & 0_{3\times 3} & C_{b}^{\mathrm{li}} & 0_{3\times 3}\\ F_{b}\left(t\right) & 0_{3\times 3} & F_{a}\left(t\right) & 0_{3\times 3} & C_{b}^{\mathrm{li}}\\ 0_{3\times 3} & I_{3\times 3} & 0_{3\times 3} & 0_{3\times 3} & 0_{3\times 3}\\ 0_{6\times 3} & 0_{6\times 3} & 0_{6\times 3} & 0_{6\times 3} & 0_{6\times 3} \end{array}\right],$$

where $I_{3}$ is the unit matrix with the dimension 3×3 and $C_{b}^{\mathrm{li}}$ is the rotation matrix from b-frame to li-frame. G(t) is the noise drive matrix. Specific expressions of $F_{a}\left(t\right)$, $F_{b}\left(t\right)$ and G(t) can be found in [26]. $W\left(t\right)={\left[\varepsilon_{s}{\left(t\right)}^{T},\nabla_{s}{\left(t\right)}^{T}\right]}^{T}$ is the system noise vector, where $\varepsilon_{s}\left(t\right)={\left[\varepsilon_{s,x}\left(t\right),\varepsilon_{s,y}\left(t\right),\varepsilon_{s,z}\left(t\right)\right]}^{T}$ is the noise of the angle drift and $\nabla_{s}\left(t\right)={\left[\nabla_{s,x}\left(t\right),\nabla_{s,y}\left(t\right),\nabla_{s,z}\left(t\right)\right]}^{T}$ is the noise of the accelerometer constant offset.

Discretize Equation (1), the state transition equation of the navigation system can be obtained as follows,

(3) $$X_{k+1}^{}=\Phi_{k}^{}X_{k}^{}+\Gamma_{k}W_{k},$$

where the subscript *k* represents the time,

(4) $$X_{k}^{}={\left[\phi_{k}^{T},\delta V_{k}^{T},\delta r_{k}^{T},\varepsilon_{k}^{T},\nabla_{k}^{T}\right]}^{T}.$$

When the discrete time step is *T*, we have

(5) $$\Phi_{k}^{}=I_{15}+F_{k}T+\frac{1}{2}F_{k}^{2}T^{2},$$

(6) $$\Gamma_{k}=T\left(I_{15}+\frac{1}{2}F_{k}T+\frac{1}{6}F_{k}^{2}T^{2}\right)G_{k},$$

where $I_{15}$ is the unit matrix with the dimension 15×15. It is generally assumed that the system noise, $W_{k}={\left[\varepsilon_{s,k}^{T},\nabla_{s,k}^{T}\right]}^{T}$, obeys the Gaussian distribution with zero mean value and the covariance matrix of $Q_{k}$.

### 2.3. Landmark Observation Equation

The effective extraction, matching, and tracking of the landmarks based on the earth image are the preconditions for the construction of the observation equation. The progress in the following aspects paves the way for the application of the proposed navigation method. First, with the increase of the number of satellites, more and more high-precision earth images can be obtained, which means that more landmarks can be observed from the missile platform. Next, there has been a variety of methods concentrating on image matching such as Harris detector [27], Scale-invariant feature transform (SIFT) algorithm [28], and speeded up robust features (SURF) algorithm [29]. These algorithms can basically achieve the fast identification of the landmark and resist the influence of light change, mist interference and other environmental changes. Finally, with the increase of computer computing speed, it has been possible to perform feature matching in real time. For example, in [30], GPU-based implementation of SURF is able to extract and match features from images with 640 × 480 resolution at 103 frames per second. In [31], an efficient FPGA-based implementation of SURF is developed to process images with 800 × 600 resolution at 60 frames per second.

**Figure 2:**
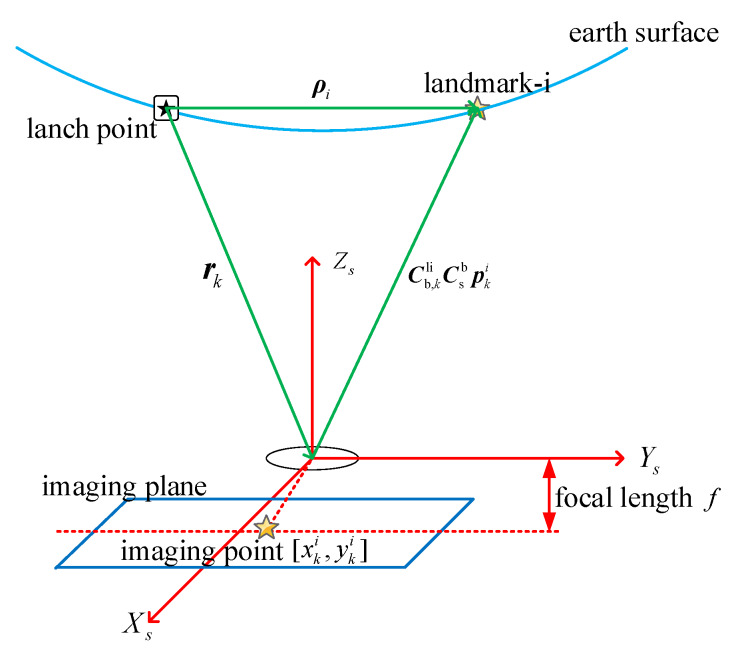
Schematic diagram of LINS observation principle.

Under the support of the above developed technologies, we establish the observation model as follows. First of all, the observation diagram of the landmark is given in Figure 2.

As Figure 2 shows, if landmark-i with known coordinate $\rho_{i}$ (expressed in li-frame) is caught through the image recognition and matching, the following equation can be derived from the geometric relationship,

(7) $$\rho_{i}=r_{k}+C_{b,k}^{\mathrm{li}}C_{s}^{b}p_{k}^{i},$$

where $p_{k}^{i}$ (expressed in s-frame) denotes the vector from the missile platform to landmark-i. Three axes of s-frame, $X_{S}$, $Y_{s}$, and $Z_{s}$, are shown in Figure 2, $r_{k}$ represents the position of the missile platform at time *k*. $C_{s}^{b}$ is the rotation matrix from s-frame to b-frame, which is related to the installation of the imaging sensor. $C_{b,k}^{\mathrm{li}}$ is the rotation matrix from b-frame to li-frame at time *k* expressed as follows,

(8) $$C_{b,k}^{\mathrm{li}}=\left[\begin{array}{ccc} \cos\varphi_{k}\cos\psi_{k} & \cos\varphi_{k}\sin\psi_{k}\sin\gamma_{k}-\sin\varphi_{k}\cos\gamma_{k} & \cos\varphi_{k}\sin\psi_{k}\cos\gamma_{k}+\sin\varphi_{k}\sin\gamma_{k}\\ \sin\varphi_{k}\cos\psi_{k} & \sin\varphi_{k}\sin\psi_{k}\sin\gamma_{k}+\cos\varphi_{k}\cos\gamma_{k} & \sin\varphi_{k}\sin\psi_{k}\cos\gamma_{k}-\cos\varphi_{k}\sin\gamma_{k}\\ -\sin\psi_{k} & \sin\gamma_{k}\cos\psi_{k} & \cos\gamma_{k}\cos\psi_{k} \end{array}\right],$$

where $\varphi_{k},\psi_{k},$ and $\gamma_{k}$ denote the pitch, yaw, and roll angles of the missile platform, respectively.

Equation (9) can be derived from Equation (7).

(9) $$p_{k}^{i}=C_{b}^{s}C_{\mathrm{li},k}^{b}\left(\rho_{i}-r_{k}\right),$$

where $C_{\mathrm{li}}^{b}$ is the rotation matrix from li-frame to b-frame and $C_{b}^{s}$ is the rotation matrix from b-frame to s-frame, and we also have

(10) $$C_{\mathrm{li}}^{b}=C_{b}^{\mathrm{li},T},$$

(11) $$C_{b}^{s}=C_{s}^{b,T}.$$

Assume that the theoretical imaging coordinate of landmark-i is $\left[x_{k}^{i},y_{k}^{i}\right]$, then the following equation holds,

(12) $$\left[\begin{array}{c} x_{k}^{i}\\ y_{k}^{i} \end{array}\right]=\left[\begin{array}{c} -f\frac{p_{k,x}^{i}}{p_{k,z}^{i}}\\ -f\frac{p_{k,y}^{i}}{p_{k,z}^{i}} \end{array}\right],$$

where $p_{k,x}^{i},p_{k,y}^{i},p_{k,z}^{i}$ are three components of $p_{k}^{i}$ and *f* is the focal length of the imaging sensor.

The above derivation proves that the imaging coordinate of landmark-i can be determined by its location and the position and attitude of the missile platform when the installation matrix of the landmark sensor, i.e., $C_{s}^{b}$ is known. However, the real state of the missile platform is unknown. However, the position output ${\hat{r}}_{k}$ and the attitude output $\zeta_{k}={\left[{\hat{\varphi }}_{k},{\hat{\psi }}_{k},{\hat{\gamma }}_{k}\right]}^{T}$ of INS can be used to obtain the estimation of $p_{k}^{i}$, ${\hat{p}}_{k}^{i}$. The expression of ${\hat{p}}_{k}^{i}$ and the corresponding estimation error, $\delta p_{k}^{i}$, can be given as follows,

(13) $${\hat{p}}_{k}^{i}=C_{b}^{s}{\hat{C}}_{\mathrm{li},k}^{b}\left(\rho_{i}-{\hat{r}}_{k}\right),$$

(14) $$\delta p_{k}^{i}\approx C_{b}^{s}h\left(\zeta_{k},\rho_{i}-{\hat{r}}_{k}\right)\theta_{k}-C_{b}^{s}{\hat{C}}_{\mathrm{li},k}^{b}\delta r_{k},$$

where ${\hat{C}}_{\mathrm{li},k}^{b}$ is the rotation matrix from li-frame to b-frame corresponding to $\zeta_{k}$, $\delta r_{k}={\left[\delta x_{k},\delta y_{k},\delta z_{k}\right]}^{T}$ is the estimation error of the missile platform position. For the vector $\omega ={\left[\omega_{x},\omega_{y},\omega_{z}\right]}^{T}$, lines 1, 2, and 3 of $h(\zeta_{k},\omega )$ are as follows, respectively.

(15) $$h{\left(\zeta_{k},\omega \right)}_{1}={\left[\begin{array}{c} -\omega_{x}\sin{\hat{\varphi }}_{k}\cos{\hat{\psi }}_{k}+\omega_{y}\cos{\hat{\varphi }}_{k}\cos{\hat{\psi }}_{k}\\ -\omega_{x}\cos{\hat{\varphi }}_{k}\sin{\hat{\psi }}_{k}-\omega_{y}\sin{\hat{\varphi }}_{k}\sin{\hat{\psi }}_{k}-\omega_{z}\cos{\hat{\psi }}_{k}\\ 0 \end{array}\right]}^{T},$$

(16) $$\begin{array}{c} h{\left(\zeta_{k},\omega \right)}_{2}=\\ {\left[\begin{array}{c} -\omega_{x}\sin{\hat{\varphi }}_{k}\sin{\hat{\psi }}_{k}\sin{\hat{\gamma }}_{k}-\omega_{x}\cos{\hat{\varphi }}_{k}\cos{\hat{\gamma }}_{k}+\omega_{y}\cos{\hat{\varphi }}_{k}\sin{\hat{\psi }}_{k}\sin{\hat{\gamma }}_{k}-\omega_{y}\sin{\hat{\varphi }}_{k}\cos{\hat{\gamma }}_{k}\\ \omega_{x}\cos{\hat{\varphi }}_{k}\cos{\hat{\psi }}_{k}\sin{\hat{\gamma }}_{k}+\omega_{y}\sin{\hat{\varphi }}_{k}\cos{\hat{\psi }}_{k}\sin{\hat{\gamma }}_{k}-\omega_{z}\sin{\hat{\psi }}_{k}\sin{\hat{\gamma }}_{k}\\ \omega_{x}\cos{\hat{\varphi }}_{k}\sin{\hat{\psi }}_{k}\cos{\hat{\gamma }}_{k}+\omega_{x}\sin{\hat{\varphi }}_{k}\sin{\hat{\gamma }}_{k}+\omega_{y}\sin{\hat{\varphi }}_{k}\sin{\hat{\psi }}_{k}\cos{\hat{\gamma }}_{k}-\omega_{y}\cos{\hat{\varphi }}_{k}\sin{\hat{\gamma }}_{k}+\omega_{z}\cos{\hat{\psi }}_{k}\cos{\hat{\gamma }}_{k} \end{array}\right]}^{T}, \end{array}$$

(17) $$h{\left(\zeta_{k},\omega \right)}_{3}={\left[\begin{array}{c} -\omega_{x}\sin{\hat{\varphi }}_{k}\sin{\hat{\psi }}_{k}\cos{\hat{\gamma }}_{k}+\omega_{x}\cos{\hat{\varphi }}_{k}\sin{\hat{\gamma }}_{k}+\omega_{y}\cos{\hat{\varphi }}_{k}\sin{\hat{\psi }}_{k}\cos{\hat{\gamma }}_{k}+\omega_{y}\sin{\hat{\varphi }}_{k}\sin{\hat{\gamma }}_{k}\\ \omega_{x}\cos{\hat{\varphi }}_{k}\cos{\hat{\psi }}_{k}\cos{\hat{\gamma }}_{k}+\omega_{y}\sin{\hat{\varphi }}_{k}\cos{\hat{\psi }}_{k}\cos{\hat{\gamma }}_{k}-\omega_{z}\sin{\hat{\psi }}_{k}\cos{\hat{\gamma }}_{k}\\ -\omega_{x}\cos{\hat{\varphi }}_{k}\sin{\hat{\psi }}_{k}\sin{\hat{\gamma }}_{k}+x\sin{\hat{\varphi }}_{k}\cos{\hat{\gamma }}_{k}-\omega_{y}\sin{\hat{\varphi }}_{k}\sin{\hat{\psi }}_{k}\sin{\hat{\gamma }}_{k}-\omega_{z}\cos{\hat{\psi }}_{k}\sin{\hat{\gamma }}_{k} \end{array}\right]}^{T}.$$

The derivation of $h(\zeta_{k},\omega )$ is given in Appendix A.

In Equation (14), $\theta_{k}={\left[\delta \varphi_{k},\delta \psi_{k},\delta \gamma_{k}\right]}^{T}$ is the attitude error angle resulted from the misalignment angle outputted by gyro. The transition equation from $\phi_{k}$ to $\theta_{k}$ is as follows,

(18) $$\theta_{k}=\frac{1}{\cos{\hat{\psi }}_{k}}\left[\begin{array}{ccc} -\cos{\hat{\varphi }}_{k}\sin{\hat{\psi }}_{k} & -\sin{\hat{\varphi }}_{k}\sin{\hat{\psi }}_{k} & -\cos{\hat{\psi }}_{k}\\ \sin{\hat{\varphi }}_{k}\cos{\hat{\psi }}_{k} & -\cos{\hat{\varphi }}_{k}\cos{\hat{\psi }}_{k} & 0\\ -\cos{\hat{\varphi }}_{k} & -\sin{\hat{\varphi }}_{k} & 0 \end{array}\right]\phi_{k}≜\Theta_{k}\phi_{k},$$

The detailed derivation of $\Theta_{k}$ can be found in [32].

According to ${\hat{p}}_{k}^{i}$ and its estimation error $\delta p_{k}^{i}$, the imaging coordinate estimation of landmark-i, ${\hat{z}}_{k}^{i}$, and its relevant estimation error, $\delta z_{k}^{i}$, can be given as follows,

(19) $${\hat{z}}_{k}^{i}=\left[\begin{array}{c} {\hat{x}}_{k}^{i}\\ {\hat{y}}_{k}^{i} \end{array}\right]=\left[\begin{array}{c} -f\frac{{\hat{p}}_{k,x}^{i}}{{\hat{p}}_{k,z}^{i}}\\ -f\frac{{\hat{p}}_{k,y}^{i}}{{\hat{p}}_{k,z}^{i}} \end{array}\right],$$

(20) $$\delta z_{k}^{i}\approx H_{C,k}^{i}\delta p_{k}^{i},$$

where

(21) $$H_{C,k}^{i}=-\frac{f}{{\hat{p}}_{k,z}^{i}}\left[\begin{array}{cc} I_{2} & \frac{{\hat{z}}_{k}^{i}}{f} \end{array}\right],$$

$I_{2}$ is the unit matrix with the dimension 2×2.

Further, the observation of landmark-i can be expressed as Equation (22).

(22) $$z_{k}^{i}=\left[\begin{array}{c} x_{k}^{i}\\ y_{k}^{i} \end{array}\right]+v_{k}^{i}={\hat{z}}_{k}^{i}+\delta z_{k}^{i}+v_{k}^{i}.$$

where $v_{k}^{i}$ is the observation noise and it is usually considered to obey Gaussian distribution with zero mean value and covariance matrix of $R_{k}^{i}$.

Based on the above equation, the implicit observation equation can be constructed as follows,

(23) $$\delta z_{k}^{i}=z_{k}^{i}-{\hat{z}}_{k}^{i}\approx \left[H_{\phi_{k}}^{i},H_{\delta r_{k}}^{i}\right]{\left[\phi_{k},\delta r_{k}\right]}^{T}+v_{k}^{i}$$

where

(24) $$H_{\phi_{k}}^{i}=H_{C,k}^{i}C_{b}^{s}h\left(\zeta_{k},\rho_{i}-{\hat{r}}_{k}\right)\Theta_{k}$$

(25) $$H_{\delta r_{k}}^{i}=-H_{C,k}^{i}C_{b}^{s}{\hat{C}}_{\mathrm{li},k}^{b}$$

So far, the imaging coordinate estimation of landmark-i, ${\hat{z}}_{k}^{i}$, has been derived through the position and attitude outputted by INS. Furthermore, the observation equation about INS output deviation, $\theta_{k}$ and $\delta r_{k}$, has been established by subtracting ${\hat{z}}_{k}^{i}$ from $z_{k}^{i}$.

### 2.4. State Estimation Process of Missile Platform

The ballistic error propagation model and the observation equation have been obtained in Section 2.2 and Section 2.3, respectively. Then, the filter algorithm of LINS can be given as follows by applying Kalman filter to the state estimation for the missile platform. The filter algorithm is as follows.

STEP1: According to the state estimation at time k−1, ${\hat{X}}_{k-1}$, and the corresponding estimation error covariance matrix $P_{k-1}$, calculate the state prediction, ${\hat{X}}_{k,k-1}$, and $P_{k,k-1}$ through the following equation.

(26) $${\hat{X}}_{k,k-1}^{}=\Phi_{k-1}^{}{\hat{X}}_{k-1}^{}$$

(27) $$P_{k,k-1}=\Phi_{k-1}^{}P_{k-1}\Phi_{k-1}^{T}+\Gamma_{k-1}W_{k-1}\Gamma_{k-1}^{T}$$

STPE2: Assume that *n* landmarks are matched at time *k*, the implicit observation equation can be obtained by computing Equations (28)–(32).

(28) $${\hat{p}}_{k}^{i}=C_{b}^{s}{\hat{C}}_{\mathrm{li},k}^{b}\left(\rho_{i}-{\hat{r}}_{k}\right)$$

(29) $${\hat{z}}_{k}^{i}=\left[\begin{array}{c} {\hat{x}}_{k}^{i}\\ {\hat{y}}_{k}^{i} \end{array}\right]=\left[\begin{array}{c} -f\frac{{\hat{p}}_{k,x}^{i}}{{\hat{p}}_{k,z}^{i}}\\ -f\frac{{\hat{p}}_{k,y}^{i}}{{\hat{p}}_{k,z}^{i}} \end{array}\right]$$

(30) $$\delta p_{k}^{i}\approx C_{b}^{s}h\left(\zeta_{k},\rho_{i}-{\hat{r}}_{k}\right)\theta_{k}-C_{b}^{s}{\hat{C}}_{\mathrm{li},k}^{b}\delta r_{k}$$

(31) $$\delta z_{k}^{i}\approx H_{C,k}^{i}\delta p_{k}^{i}$$

(32) $$Z_{k}=\left[\begin{array}{c} \delta z_{k}^{1}\\ \vdots \\ \delta z_{k}^{n} \end{array}\right]$$

STEP3: Calculate ${\hat{X}}_{k}^{}$ and corresponding $P_{k}$ through Equations (33)–(37). Estimate the missile platform state by compensating ${\hat{X}}_{k}^{}$ to the INS output. Set k=k+1 and return to STEP1.

(33) $$H_{k}=\left[\begin{array}{cccc} H_{\phi_{k}}^{1} & 0_{2\times 3} & H_{\delta r_{k}}^{1} & 0_{2\times 6}\\ \vdots & \vdots & \vdots & \vdots \\ H_{\phi_{k}}^{n} & 0_{2\times 3} & H_{\delta r_{k}}^{n} & 0_{2\times 6} \end{array}\right]$$

(34) $$R_{k}=\left[\begin{array}{ccc} R_{k}^{1} & & \\ & \ddots & \\ & & R_{k}^{n} \end{array}\right]$$

(35) $$K_{k}=P_{k,k-1}H_{k}^{T}{\left(H_{k}P_{k,k-1}H_{k}^{T}+R_{k}\right)}^{T}$$

(36) $${\hat{X}}_{k}^{}={\hat{X}}_{k,k-1}^{}+K_{k}\left(Z_{k}-H_{k}{\hat{X}}_{k,k-1}^{}\right)$$

(37) $$P_{k}=\left(I_{15}-K_{k}H_{k}\right)P_{k,k-1}{\left(I_{15}-K_{k}H_{k}\right)}^{T}+K_{k}R_{k}K_{k}^{T}$$

## 3. Observability Analysis

The observability is the premise of the effective implementation for the navigation scheme and the filtering algorithm. Therefore, in this section, the observability of the proposed navigation system is analyzed from two aspects, the physical observability and the mathematical observability. The so-called physical observability refers to the visibility of the landmark to the missile. The mathematical observability reflects the ability of the navigation system to achieve the optimal estimation of the system state by combining the measurements with the system state model [33].

### 3.1. Physical Observability

**Figure 3:**
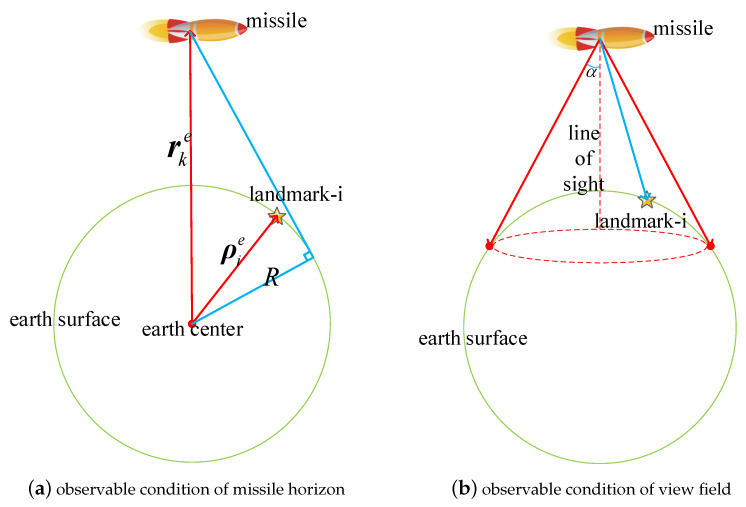
Physical observability analysis diagram.

In order to make the landmark visible to the landmark sensor installed on the missile platform, the conditions shown in Figure 3 should be satisfied. First, the landmark should be within the horizon of the missile position (seen as Figure 3a). Second, the landmark should be located in the view field of the landmark sensor (seen as Figure 3b).

In Figure 3a, $\rho_{i}^{e}$ and $r_{k}^{e}$ are the position vectors of landmark-i and the missile platform expressed in the earth fixed coordinate frame, respectively. *R* denotes the earth radius. In order to satisfy the physical condition, the following formula should hold.

(38) $$\arccos\left(\frac{\rho_{i}^{e,T}\cdot r_{k}^{e}}{∥\rho_{i}^{e}∥∥r_{k}^{e}∥}\right)<\arccos\left(\frac{R}{∥r_{k}^{e}∥}\right),$$

where $∥\cdot ∥$ denotes the 2-norm of a vector.

Here, we assume that the earth is regarded as a standard sphere, which may lead to the erroneous judgement of the observability. For the sake of safety, *R* can be taken as the shortest distance from the center of the earth to its surface.

Figure 3b shows that the landmark should be in the view field of the landmark sensor. If the field angle of the landmark sensor is 2α, then in order to meet the field condition, the following equation should be satisfied,

(39) $$\arccos(\frac{\left[0,0,1\right]\cdot p_{k}^{i}}{∥p_{k}^{i}∥})<\alpha .$$

In addition to the two conditions shown in Figure 3, the landmark may not be observed due to the absence of visible light. This means that the proposed navigation system may only be applicable to missiles launched in the daytime. However, it should be noted that due to the strong radiation of the atmospheric background during the day, the difficulty of the CINS in the star observation limits its application in the daytime launched missiles [34,35]. The proposed system may be an alternative to the CINS under the strong visible light. In addition, the number of observable landmarks may be reduced when the missile passes through the desert, ocean, and areas covered by clouds or fog. However, the following simulation shows that even though the number of matched landmarks is small, the LINS can still achieve high-accuracy state estimation.

### 3.2. Mathematical Observability

For the sake of simplicity, assume that only one landmark is observed, and then the mathematical observability matrix [36] of LINS can be given as follows,

(40) $$M=\left[\begin{array}{c} H\\ H\Phi \\ \vdots \\ {H\Phi }_{}^{14} \end{array}\right],$$

where

(41) $$H=[H_{\phi_{}}^{},0_{2\times 3},H_{\delta r_{}}^{},0_{2\times 6}],$$

(42) $$\Phi =I_{15}+FT+\frac{1}{2}F_{}^{2}T^{2},$$

The symbols used in Equations (40)–(42) have been defined above. The fact that the time subscript *k* is omitted means that symbols here are independent from time. Then

(43) $$\begin{array}{cc} \; & H\Phi =H\left(I_{15}+FT+\frac{1}{2}F_{}^{2}T^{2}\right)=H+T\mathrm{HF}+\frac{1}{2}T^{2}{\mathrm{HF}}_{}^{2}=\\ \; & \left[\begin{array}{ccccc} H_{\phi }^{}+\frac{1}{2}T^{2}H_{\delta r}^{}F_{b}, & TH_{\delta r}^{}, & H_{\delta r}^{}\left(I_{3}+\frac{1}{2}T^{2}F_{a}\right), & TH_{\phi }^{}C_{b}^{\mathrm{li}}, & \frac{1}{2}T^{2}H_{\delta r}^{}C_{b}^{\mathrm{li}} \end{array}\right]. \end{array}$$

Obviously, HΦ has full column rank, so M also has full column rank. Therefore, the proposed navigation system is fully observable when the number of observable landmarks is not less than 1.

## 4. Simulation

In order to verify the advantages of LINS, the traditional CINS and the deeply integrated CINS proposed in [15] are used as the control group of the comparative simulation experiment. Parameter settings of the missile trajectory, the landmark sensor and the INS output are shown in Table 1, Table 2 and Table 3, in which settings of the ballistic parameter and INS output parameter have referred to [37].

In addition, set the attitude estimation accuracy of the star sensor used in CINS as 8 arc seconds, the measurement accuracy of the altimeter in the deeply integrated CINS as 50 m and the rotation matrix from s-frame to b-frame as follows,

(44) $$C_{s}^{b}=\left[\begin{array}{ccc} 1 & 0 & 0\\ 0 & 0 & -1\\ 0 & -1 & 0 \end{array}\right]$$

The filter periods of the three navigation systems, i.e., the time intervals between two adjacent state estimation of the filter, are set to 0.1 s. Then, 200 landmarks are randomly generated in the region with the longitude $\in \left[116.34,\;188.57\right]$ and the latitude ∈[14.028,57.169]. In order to test the state estimation performance of the proposed algorithm under different number of measurements, the following two cases are considered.

Case 1: LINS can apply the landmarks to the state estimation as long as they are observed.

Case 2: Up to three observable landmarks are randomly selected and used for missile state estimation.

Case 1 is a simulation of the actual navigation scene, whereas Case 2 tests the performance of LINS when the number of observable landmarks is small. It should be noted that in Case 2, LINS is provided with the same or less measurements as the deeply integrated CINS which requires at least two stars to obtain the attitude estimation and additional altitude observations to estimate the missile position.

**Figure 4:**
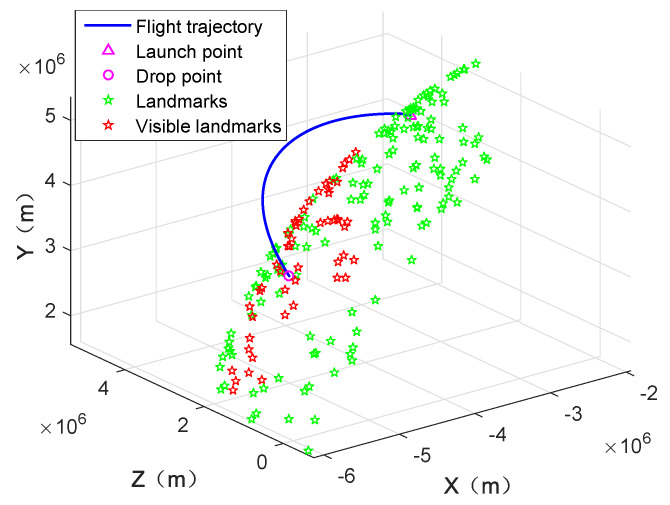
Simulated missile trajectory and landmarks.

**Figure 5:**
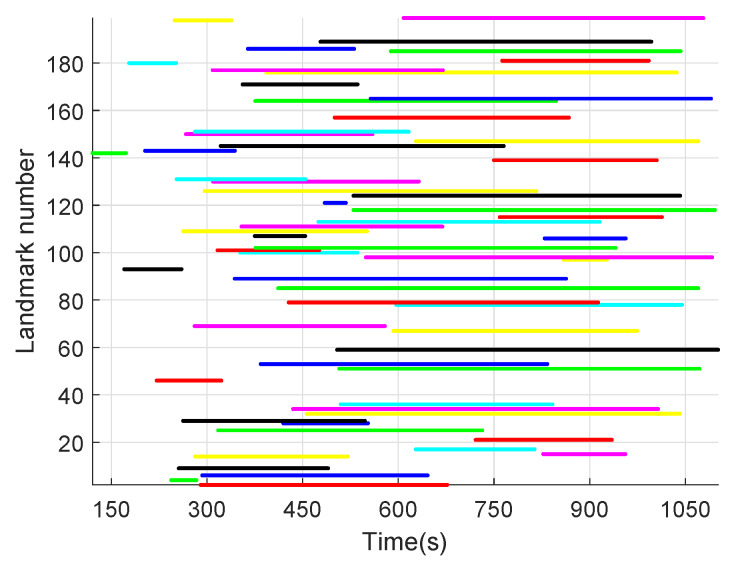
Landmark observation episodes.

**Figure 6:**
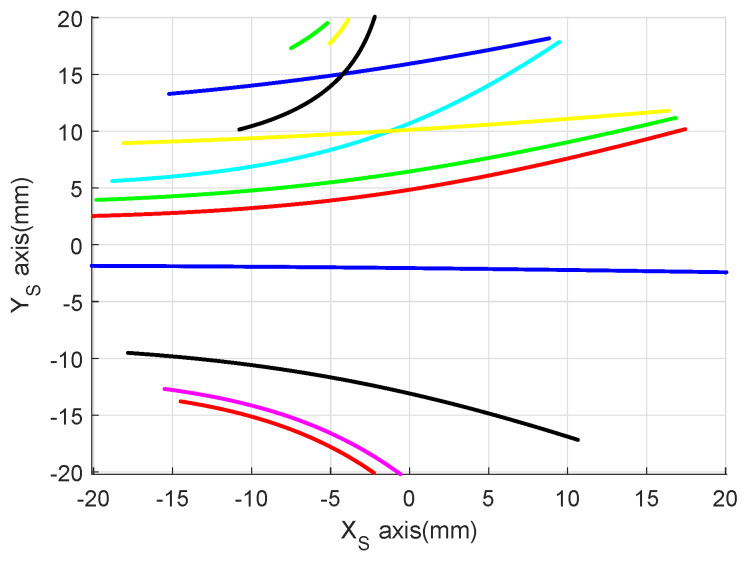
Imaging track of observable landmarks.

Figure 4 shows the generated trajectory of the missile in the earth inertial frame, the randomly generated landmarks (represented by green pentagram), and the observed landmarks (represented by red pentagram). Figure 5 displays the observation episodes of the landmarks. When landmarks-i is observed at time k, the corresponding location is marked with the real point, otherwise there is no mark. Figure 6 illustrates the imaging track of the observable landmarks constituted composed of imaging coordinates at different time.

**Figure 7:**
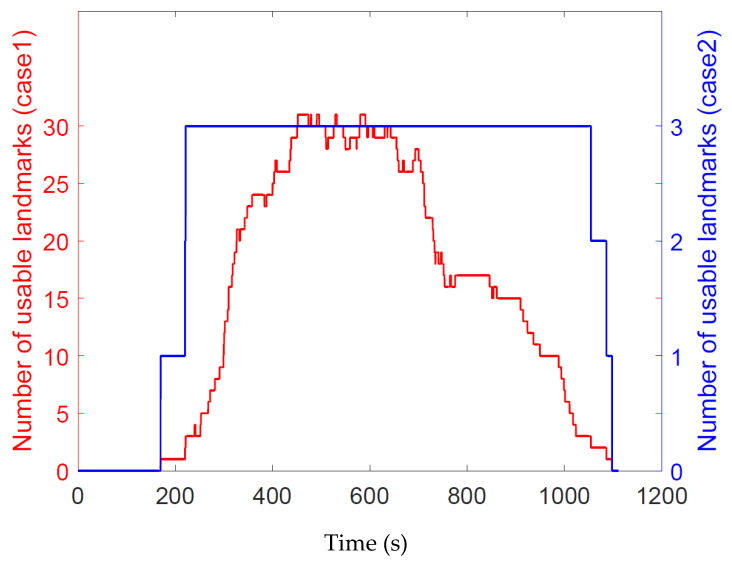
Number of the visible landmarks.

Figure 7 gives the number of landmarks that can be used for the state estimation of the missile platform under two cases. From Figure 7, it can be seen that the number of the observable landmarks is small or even zero during the early and late flight stages due to the limited flight altitude and the landmark density. When the number of the observable landmarks is 0, the proposed navigation system cannot work and the system output is consistent with that of the INS. In the practical application, the suitable storage of the geodetic image may avoid the situation of too few visible landmarks. Meanwhile, because of the number limitation, under Case 2, the maximum number of landmarks that can be used for state estimation is 3.

**Figure 8:**
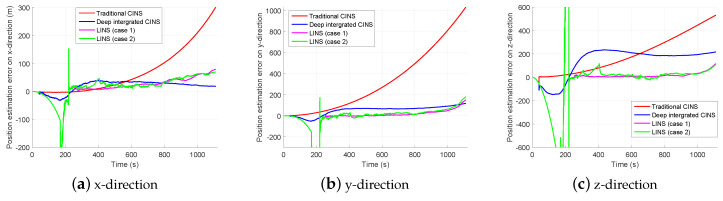
Position estimation errors of different navigation systems.

**Figure 9:**
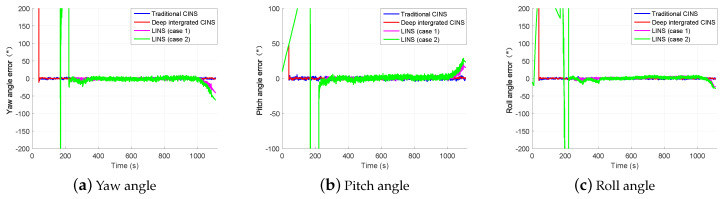
Attitude estimation errors of different navigation systems.

Figure 8 and Figure 9 show the position and attitude estimation errors given by CINS of two different combination modes and by LINS under two cases with different number of usable landmarks. As seen from Figure 7 and Figure 8, the position estimation accuracy of the proposed navigation system increases rapidly with the number of observable landmarks rising. Even when the maximum number of landmarks available for state estimation is 3, LINS outperforms CINS in the position estimation of the missile platform. However, when the number is not limited, LINS behaves more stable in position estimation.

Similarly, according to Figure 7 and Figure 9, it can be seen that the accuracy of attitude estimation obtained by LINS increases when the number of observable landmarks rises. LINS performs satisfactory in attitude estimation even when the maximum number of available landmarks is 3.

Table 4 compares the root mean square error (RMSE) of the position and attitude estimation given by CINS of two combination modes and LISN under two cases of different number of usable landmarks. It should be noted that datas in Table 4 are the average results of 100 Monte Carlo experiments when the number of observable landmarks exceeds 3. As seen from the table, LINS performs better than CINS in the position estimation even when the maximum number of landmarks available for position estimation is reduced to 3. Under Case 2, the position estimation error of LINS is 80.90% and 61.55% lower than that of the traditional CINS and the deeply integrated CINS, respectively. It means that LINS performs more satisfactory than CINS in the position estimation of the missile platform. In addition, in the attitude estimation, the proposed system is slightly inferior to the CINS, but its attitude estimation accuracy remains high. Under two cases of different number of available landmarks, the average RMSE of attitude estimation that LINS obtains by 100 simulation experiments is 2.90 arc second and 1.67 arc second, respectively.

## 5. Conclusions

In order to deal with difficulties of CINS in the position estimation, LINS is designed in this paper. In the proposed method, the implicit observation equation of the INS output deviation is derived firstly. Next, the physical observability of the landmark and the mathematical observability of the LINS are analyzed. Theoretical analysis shows that the proposed navigation system is fully observable in mathematics. Compared with the traditional CINS and the deeply integrated CINS, the simulation results demonstrate that position estimation RMSE of the proposed navigation method is 80.90% and 61.55% lower than that of the other two systems, respectively, even when the maximum number of landmarks that can be used for state estimation is 3. Although LINS does not perform as excellent as the other two navigation methods in the attitude estimation, its estimation accuracy still maintains high.

It should be pointed out that the influence of the atmospheric refraction, the aerodynamics, etc. on the accuracy of landmark observation is not considered in this paper. In the future research, these observation errors will be dealt with. In addition, the number of observable landmarks may be small under the condition that the missile passes through the ocean, the desert, and areas covered by clouds or fog. The estimation of the missile platform with smaller number of observable landmarks needs also to be studied in the future.

## Figures and Tables

**Table 1:** Parameters of missile trajectory.

Initial Longitude (${}^{\circ }$)	Initial Latitude (${}^{\circ }$)	Initial Velocity (m$\cdot s^{-1}$)	Initial Pitch Angle (${}^{\circ }$)
116.34	39.98	${\left[355.49,\;0,\;0\right]}^{T}$	90
**Vertical Rise Time (s)**	**Ending Time of the** **Powered Phase Turn (s)**	**Time of the** **Engine Shutting off (s)**	**Total Flying Time (s)**
10	60	160	1110

**Table 2:** Parameters of landmark sensor.

α (${}^{\circ }$)	*f* (mm)	$R_{k}^{i}$ ($um^{2}$)	Sampling Period (s)
40	35	1	0.1

**Table 3:** Parameters of Inertial Navigation System (INS) output.

Gravity Acceleration $g\;(m^{2}\cdot s^{-2})$	$\varepsilon_{k}$ (${}^{\circ }\cdot h^{-1}$)	$\nabla_{k}$ (ug)	$E(\varepsilon_{s,k}^{2})$ (${{}^{\circ }}^{2}\cdot h^{-2}$)	$E(\nabla_{s,k}^{2})$ ($ug^{2}$)
9.78	${\left[1,\;1,\;1\right]}^{T}$	${\left[100,\;100,\;100\right]}^{T}$	diag([0.25,0.25,0.25])	diag([2500,2500,2500])

**Table 4:** State estimation accuracy of different navigation systems.

Navigation System	Poisition Estimation RMSE (m)				Attitude Estimation RMSE (″)			
	x	y	z	Total	x	y	z	Total
Traditional CINS	87.29	363.25	220.56	250.48	0.81	0.79	0.83	0.81
Deeply integrated CINS	32.71	67.68	201.91	124.39	1.33	1.27	1.31	1.30
LINS (case 2)	37.66	23.70	69.89	47.83	2.67	2.51	3.43	2.90
LINS (case 1)	27.82	13.75	11.91	19.19	1.46	0.82	2.35	1.67

## Abbreviations

The following abbreviations are used in this manuscript.

INS	Inertial Navigation System
GNSS	Global Navigation Satellite System
CNS	Celestial Navigation System
CINS	Celestial-Inertial Navigation System
LINS	Landmark-based Inertial Navigation System
li-frame	launch-point inertial coordinate frame
b-frame	missile body coordinate frame
s-frame	sensor coordinate frame
RMSE	root mean square error

## Appendix A

Now, we have the real attitude angle of the missile platform, $\vartheta ={\left[\varphi_{k},\psi_{k},\gamma_{k}\right]}^{T}$, the estimation of the angle, $\zeta_{k}={\left[{\hat{\varphi }}_{k},{\hat{\psi }}_{k},{\hat{\gamma }}_{k}\right]}^{T}$ and the corresponding estimation error $\theta_{k}={\left[\delta \varphi_{k},\delta \psi_{k},\delta \gamma_{k}\right]}^{T}$. The rotation matrices from li-frame to b-frame related to ϑ and $\zeta_{k}$ are shown in Equations (A1) and (A2), respectively.

(A1) $$C=\left[\begin{array}{ccc} \cos\varphi_{k}\cos\psi_{k} & \sin\varphi_{k}\cos\psi_{k} & -\sin\psi_{k}\\ \cos\varphi_{k}\sin\psi_{k}\sin\gamma_{k}-\sin\varphi_{k}\cos\gamma_{k} & \sin\varphi_{k}\sin\psi_{k}\sin\gamma_{k}+\cos\varphi_{k}\cos\gamma_{k} & \sin\gamma_{k}\cos\psi_{k}\\ \cos\varphi_{k}\sin\psi_{k}\cos\gamma_{k}+\sin\varphi_{k}\sin\gamma_{k} & \sin\varphi_{k}\sin\psi_{k}\cos\gamma_{k}-\cos\varphi_{k}\sin\gamma_{k} & \cos\gamma_{k}\cos\psi_{k} \end{array}\right],$$

(A2) $$\hat{C}=\left[\begin{array}{ccc} \cos{\hat{\varphi }}_{k}\cos{\hat{\psi }}_{k} & \sin{\hat{\varphi }}_{k}\cos{\hat{\psi }}_{k} & -\sin{\hat{\psi }}_{k}\\ \cos{\hat{\varphi }}_{k}\sin{\hat{\psi }}_{k}\sin{\hat{\gamma }}_{k}-\sin{\hat{\varphi }}_{k}\cos{\hat{\gamma }}_{k} & \sin{\hat{\varphi }}_{k}\sin{\hat{\psi }}_{k}\sin{\hat{\gamma }}_{k}+\cos{\hat{\varphi }}_{k}\cos{\hat{\gamma }}_{k} & \cos{\hat{\psi }}_{k}\sin{\hat{\gamma }}_{k}\\ \cos{\hat{\varphi }}_{k}\sin{\hat{\psi }}_{k}\cos{\hat{\gamma }}_{k}+\sin{\hat{\varphi }}_{k}\sin{\hat{\gamma }}_{k} & \sin{\hat{\varphi }}_{k}\sin{\hat{\psi }}_{k}\cos{\hat{\gamma }}_{k}-\cos{\hat{\varphi }}_{k}\sin{\hat{\gamma }}_{k} & \cos{\hat{\psi }}_{k}\cos{\hat{\gamma }}_{k} \end{array}\right].$$

Here, $C_{\mathrm{li},k}^{b}$ and ${\hat{C}}_{\mathrm{li},k}^{b}$ are marked as C and $\hat{C}$. For the vector $\omega ={\left[\omega_{x},\omega_{y},\omega_{z}\right]}^{T}$ expressed in the li-frame, the corresponding vector (expressed in b-frame) rotated through C and $\hat{C}$ can be derived as given in Equations (A3) and (A4).

(A3) w=Cω,

(A4) $$\hat{w}=\hat{C}\omega ,$$

Next, our task is linearly representing δw through $\theta_{k}$, where δw is the estimation error of $\hat{w}$ as shown in (A5).

(A5) $$\delta w=w-\hat{w},$$

We have

(A6) $$\left\{\begin{array}{c} \varphi_{k}={\hat{\varphi }}_{k}+\delta \varphi_{k}\\ \psi_{k}={\hat{\psi }}_{k}+\delta \psi_{k}\\ \gamma_{k}={\hat{\gamma }}_{k}+\delta \gamma_{k} \end{array}\right..\;$$

When $\delta \varphi_{k}$ is small, the following equation holds,

(A7) $$\sin\left(\delta \varphi_{k}\right)=\delta \varphi_{k},\cos\left(\delta \varphi_{k}\right)=1.$$

Therefore,

(A8) $$\left\{\begin{array}{c} \sin\left(\varphi_{k}\right)=\sin\left({\hat{\varphi }}_{k}+\delta \varphi_{k}\right)=\sin{\hat{\varphi }}_{k}+\cos{\hat{\varphi }}_{k}\cdot \delta \varphi_{k}\\ \cos\left(\varphi_{k}\right)=\cos\left({\hat{\varphi }}_{k}+\delta \varphi_{k}\right)=\cos{\hat{\varphi }}_{k}-\sin{\hat{\varphi }}_{k}\cdot \delta \varphi_{k} \end{array}.\right.$$

The law in (A8) is also applicable to the other two attitude angles when $\delta \psi_{k}$ and $\delta \gamma_{k}$ are small.

By substituting (A6) and (A8) into (A1), (A9) can be derived.

(A9) $$C=\hat{C}+{\left[J_{1},J_{2},J_{3}\right]}^{T},$$

where

(A10) $$J_{1}={\left[\begin{array}{c} -\sin{\hat{\varphi }}_{k}\cos{\hat{\psi }}_{k}\cdot \delta \varphi_{k}-\cos{\hat{\varphi }}_{k}\sin{\hat{\psi }}_{k}\cdot \delta {\hat{\psi }}_{k}\\ \cos{\hat{\varphi }}_{k}\cos{\hat{\psi }}_{k}\cdot \delta \varphi_{k}-\sin{\hat{\varphi }}_{k}\sin{\hat{\psi }}_{k}\cdot \delta {\hat{\psi }}_{k}\\ \cos{\hat{\psi }}_{k}\cdot \delta {\hat{\psi }}_{k} \end{array}\right]}^{T},$$

(A11) $$\begin{array}{cc} \; & J_{2}={\left[\begin{array}{c} -\sin{\hat{\varphi }}_{k}\sin{\hat{\psi }}_{k}\sin{\hat{\gamma }}_{k}\cdot \delta \varphi_{k}+\cos{\hat{\varphi }}_{k}\cos{\hat{\psi }}_{k}\sin{\hat{\gamma }}_{k}\cdot \delta {\hat{\psi }}_{k}\\ \cos{\hat{\varphi }}_{k}\sin{\hat{\psi }}_{k}\sin{\hat{\gamma }}_{k}\cdot \delta \varphi_{k}+\sin{\hat{\varphi }}_{k}\cos{\hat{\psi }}_{k}\sin{\hat{\gamma }}_{k}\cdot \delta {\hat{\psi }}_{k}\\ -\sin{\hat{\psi }}_{k}\sin{\hat{\gamma }}_{k}\cdot \delta {\hat{\psi }}_{k}+\cos{\hat{\psi }}_{k}\cos{\hat{\gamma }}_{k}\cdot \delta {\hat{\gamma }}_{k} \end{array}\right]}^{T}\\ \; & +{\left[\begin{array}{c} \cos{\hat{\varphi }}_{k}\sin{\hat{\psi }}_{k}\cos{\hat{\gamma }}_{k}\cdot \delta {\hat{\gamma }}_{k}-\cos{\hat{\varphi }}_{k}\cos\hat{\gamma }\cdot \delta \varphi_{k}+\sin{\hat{\varphi }}_{k}\sin{\hat{\gamma }}_{k}\cdot \delta {\hat{\gamma }}_{k}\\ \sin{\hat{\varphi }}_{k}\sin{\hat{\psi }}_{k}\cos{\hat{\gamma }}_{k}\cdot \delta {\hat{\gamma }}_{k}-\sin{\hat{\varphi }}_{k}\cos{\hat{\gamma }}_{k}\cdot \delta \varphi_{k}-\cos{\hat{\varphi }}_{k}\sin{\hat{\gamma }}_{k}\cdot \delta {\hat{\gamma }}_{k}\\ 0 \end{array}\right]}^{T}, \end{array}$$

(A12) $$\begin{array}{cc} \; & J_{3}={\left[\begin{array}{c} -\sin{\hat{\varphi }}_{k}\sin{\hat{\psi }}_{k}\cos{\hat{\gamma }}_{k}\cdot \delta \varphi_{k}+\cos{\hat{\varphi }}_{k}\cos{\hat{\psi }}_{k}\cos{\hat{\gamma }}_{k}\cdot \delta {\hat{\psi }}_{k}\\ \cos{\hat{\varphi }}_{k}\sin{\hat{\psi }}_{k}\cos{\hat{\gamma }}_{k}\cdot \delta \varphi_{k}+\sin{\hat{\varphi }}_{k}\cos{\hat{\psi }}_{k}\cos{\hat{\gamma }}_{k}\cdot \delta {\hat{\psi }}_{k}\\ -\sin{\hat{\psi }}_{k}\cos{\hat{\gamma }}_{k}\cdot \delta {\hat{\psi }}_{k}-\cos{\hat{\psi }}_{k}\sin{\hat{\gamma }}_{k}\cdot \delta {\hat{\gamma }}_{k} \end{array}\right]}^{T}\\ \; & +{\left[\begin{array}{c} -\cos{\hat{\varphi }}_{k}\sin{\hat{\psi }}_{k}\sin{\hat{\gamma }}_{k}\cdot \delta {\hat{\gamma }}_{k}+\cos{\hat{\varphi }}_{k}\sin{\hat{\gamma }}_{k}\cdot \delta \varphi_{k}+\sin{\hat{\varphi }}_{k}\cos{\hat{\gamma }}_{k}\cdot \delta {\hat{\gamma }}_{k}\\ -\sin{\hat{\varphi }}_{k}\sin{\hat{\psi }}_{k}\sin{\hat{\gamma }}_{k}\cdot \delta {\hat{\gamma }}_{k}+\sin{\hat{\varphi }}_{k}\sin{\hat{\gamma }}_{k}\cdot \delta \varphi_{k}-\cos{\hat{\varphi }}_{k}\cos{\hat{\gamma }}_{k}\cdot \delta {\hat{\gamma }}_{k}\\ 0 \end{array}\right]}^{T}. \end{array}$$

In (A10)–(A12), the higher-order small quantities are ignored.

Combining Equations (A3)–(A5) and (A9), we can obtain that

(A13) $$\delta w={\left[J_{1},J_{2},J_{3}\right]}^{T}\omega .$$

Further, (A13) can be represented as follows,

(A14) $$\delta w=h\left(\zeta_{k},\omega \right)\theta_{k},$$

where the expression of $h(\zeta_{k},\omega )$ has been given in (15)–(17).

## References

[1] Ghanbarpour H. (2014). A back-propagation approach to compensate velocity and position errors in an integrated inertial/celestial navigation system using unscented Kalman filter. Proc. Inst. Mech. Eng. Part J. Aerosp. Eng..

[2] Li J., Jing Z., Zhang X., Zhang J., Li J., Gao S. (2018). Optimization design method of a new stabilized platform based on missile-borne semi-strap-down inertial navigation system. Sensors.

[3] Dai D., Wang X., Zhan D., Huang J. (2014). An improved method for dynamic measurement of deflections of the vertical based on the maintenance of attitude reference. Sensors.

[4] Zhang H., Zheng W., Tang G. (2012). Stellar/inertial integrated guidance for responsive launch vehicles. Aerosp. Sci. Technol..

[5] Yang Y., Zhang C., Lu J. (2017). Local observability analysis of star sensor installation errors in a SINS/CNS integration system for near-earth flight vehicles. Sensors.

[6] Yang S., Yang G., Zhu Z., Li J. (2016). Stellar Refraction-Based SINS/CNS Integrated Navigation System for Aerospace Vehicles. J. Aerosp. Eng..

[7] He Z., Wang X., Fang J. (2014). An innovative high-precision SINS/CNS deeply integrated navigation scheme for the Mars rover. Aerosp. Sci. Technol..

[8] Wu X., Wang X. (2011). A SINS/CNS deeply integrated navigation method based on mathematical horizon reference. Aircr. Eng. Aerosp. Technol..

[9] Ning X., Liu L. (2014). A two-mode INS/CNS navigation method for lunar rovers. IEEE Trans. Instrum. Meas..

[10] Wang R., Xiong Z., Liu J., Shi L. (2016). A new tightly-coupled INS/CNS integrated navigation algorithm with weighted multi-stars observations. Proc. Inst. Mech. Eng. Part J. Aerosp. Eng..

[11] Wang R., Xiong Z., Liu J., Shi L. (2017). A robust astro-inertial integrated navigation algorithm based on star-coordinate matching. Aerosp. Sci. Technol..

[12] Ning X., Yuan W., Liu Y. (2019). A tightly coupled rotational SINS/CNS integrated navigation method for aircraft. J. Syst. Eng. Electron..

[13] Wang D., Lv H., An X., Wu J. (2018). A high-accuracy constrained SINS/CNS tight integrated navigation for high-orbit automated transfer vehicles. Acta Astronaut..

[14] Ning X., Gui M., Xu Y., Bai X., Fang J. (2016). INS/VNS/CNS integrated navigation method for planetary rovers. Aerosp. Sci. Technol..

[15] Yang L., Li B., Ge L. (2015). A novel SINS/CNS integrated navigation algorithm used in a ballistic missile. Int. J. Secur. Appl..

[16] Atiya S., Hager G.D. (1993). Real-time vision-based robot localization. IEEE Trans. Robot. Autom..

[17] Betke M., Gurvits L. (1997). Mobile robot localization using landmarks. IEEE Trans. Robot. Autom..

[18] Chatterji G., Menon P., Sridhar B. (1997). GPS/machine vision navigation system for aircraft. IEEE Trans. Aerosp. Electron. Syst..

[19] Scaramuzza D., Fraundorfer F. (2011). Visual odometry [tutorial]. IEEE Robot. Autom. Mag..

[20] Borenstein J., Everett H., Feng L. (1996). Where am I? Sensors and methods for mobile robot positioning. Univ. Mich..

[21] Johnson A.E., Cheng Y., Montgomery J.F., Trawny N., Zheng J.X. (2015). Real-Time Terrain Relative Navigation Test Results from a Relevant Environment for Mars Landing. AIAA Guidance, Navigation, and Control Conference.

[22] Mcgee T.G., Rosendall P.E., Hill A. (2015). APLNav: Development Status of an Onboard Passive Optical Terrain Relative Navigation System. AIAA Guidance, Navigation, and Control Conference.

[23] Xu C., Wang D., Huang X. (2016). Landmark-based autonomous navigation for pinpoint planetary landing. Adv. Space Res..

[24] Hou B., Wang J., Zhou H., He Z. (2019). Autonomous navigation method of flight around Mars based on landmark image. Control Theory Appl..

[25] Kim Y., Hwang D.-H. (2016). Vision/INS integrated navigation system for poor vision navigation environments. Sensors.

[26] Quan W., Liu B., Gong X., Fang J. (2015). INS/CNS/GNSS Integrated Navigation Technology.

[27] Harris C., Stephens M. (1988). A combined corner and edge detector. Alvey Vision Conference.

[28] Lowe D.G. (2004). Distinctive Image Features from Scale-Invariant Keypoints. Int. J. Comput. Vis..

[29] Herbert B., Andreas E., Tinne T., Luc V. (2008). Speeded-up robust features. Comput. Vis. Image Underst..

[30] Cornelis N., Gool L.V. (2008). Fast scale invariant feature detection and matching on programmable graphics hardware. Proceedings of the IEEE Computer Society Conference on Computer Vision & Pattern Recognition Workshops.

[31] Zhao J., Huang X., Massoud Y. (2014). An efficient real-time FPGA implementation for object detection. Proceedings of the 2014 IEEE 12th International New Circuits and Systems Conference (NEWCAS).

[32] Deng H., Liu G.B., Chen H.M., Liu Z.G. (2011). Deduction and simulation of angular error relationship in “SINS/CNS” integrated navigation system. J. Astronaut..

[33] Li Y., Zhang A. (2019). Observability analysis and autonomous navigation for two satellites with relative position measurements. Acta Astronaut..

[34] Wang W., Wei X., Li J., Wang G. (2017). Noise suppression algorithm of short-wave infrared star image for daytime star sensor. Infrared Phys. Technol..

[35] Dai D., Tan W., Wu W., Wang X., Qin S. An Optimal Tightly-coupled Stellar/inertial Integrated Navigation Method for Daytime Application. Proceedings of the 2018 DGON Inertial Sensors and Systems (ISS).

[36] Chen Z., Jiang K., Hung J.C. Local observability matrix and its application to observability analyses. Proceedings of the IECON’90: 16th Annual Conference of IEEE Industrial Electronics Society.

[37] Hou B., He Z., Li D., Zhou H., Wang J. (2018). Maximum correntropy unscented kalman filter for ballistic missile navigation system based on SINS/CNS deeply integrated mode. Sensors.

